# Psychometric evaluation and translation of the Persian version of the Organizational Silence Behavior Scale (OSBS-P) for clinical nurses

**DOI:** 10.1371/journal.pone.0314155

**Published:** 2024-12-30

**Authors:** Alireza Mirzaei, Mobina Jamshidinia, Mehrzad Aghabarari, Pouya Dolat Abadi, Reza Nemati-Vakilabad

**Affiliations:** 1 Department of Emergency Nursing, School of Nursing and Midwifery, Ardabil University of Medical Sciences, Ardabil, Iran; 2 Student Research Committee, School of Nursing and Midwifery, Guilan University of Medical Sciences, Rasht, Iran; 3 Student Research Committee, School of Nursing and Midwifery, Tehran University of Medical Sciences, Tehran, Iran; 4 Department of Medical-Surgical Nursing, School of Nursing and Midwifery, Ardabil University of Medical Sciences, Ardabil, Iran; Ahvaz Jundishapur University: Ahvaz Jondishapour University of Medical Sciences, ISLAMIC REPUBLIC OF IRAN

## Abstract

**Background:**

Healthcare professionals have a crucial responsibility to provide optimal patient care. However, maintaining silence within an organization can often lead to ethical dilemmas and negatively impact the quality of care. Healthcare professionals must speak up and ensure their concerns are heard and addressed to promote a safe and ethical healthcare environment. This study aimed to translate and assess the psychometric properties of the Persian version of the Organizational Silence Behavior Scale (OSBS-P) for clinical nurses.

**Methods:**

A methodological study was conducted on 338 clinical nurses to evaluate the psychometric properties of the Persian version of the 32-item OSBS-P. A multilevel approach was used: forward-backward translation, face and content validity, construct validity (confirmatory factor analysis), and reliability (internal consistency and stability) of the Persian version of the OSBS-P were analyzed, respectively.

**Results:**

The psychometric evaluation demonstrated that the Persian version of OSBS-P is not only understandable but also retains the conceptual integrity of the original English version. Confirmatory factor analysis validated its alignment with the proposed four-factor model, indicating robust construct validity. The OSBS-P’s reliability was excellent, with a Cronbach’s alpha coefficient of α = 0.969, and stability was confirmed with an intraclass correlation coefficient (ICC) of 0.951 (95% CI, 0.919–0.945).

**Conclusion:**

The OSBS-P is a valid tool for evaluating the organizational silence behavior of clinical nurses.

## Introduction

Improving healthcare systems is essential globally as they continually evolve to meet patient expectations, enhance population health, and improve the quality of care [1]. Effective communication and appropriate feedback mechanisms are vital in achieving these goals, especially among healthcare professionals who must work cohesively. Nurses, in particular, are pivotal in providing quality care and ensuring patient safety; their insights and feedback are crucial for the success of healthcare systems [2, 3].

Numerous literature reviews have shown that healthcare team members often refrain from addressing issues concerning patient safety and unprofessional behavior until they escalate into serious problems [2, 4–6]. This reluctance to speak up can harm the organization [7] and diminish commitment to core values [8]. Ethical concerns are a primary reason for nurses’ reluctance to voice their opinions [9], and feelings of being unwelcome or excluded can further contribute to this silence [7]. Other factors include perceiving one’s role as passive [10, 11], a lack of organizational identification [12], the integrity of leaders, and concerns about negatively impacting one’s academic career [13].

The importance of psychometric research in healthcare settings cannot be overstated. Several theories have been proposed to elucidate organizational silence, with Organizational Culture and Communication Theory being the most prominent. According to Organizational Culture Theory, an organization’s culture significantly impacts employees’ inclination to voice their opinions. A culture that promotes open dialogue creates an environment where employees feel comfortable expressing their concerns without fear of reprisal. Conversely, a culture discouraging dissenting views leads to heightened silence among team members [14]. Communication Theory underscores the significance of effective communication channels in addressing organizational silence. Studies have demonstrated that effective communication among healthcare professionals reduces errors and improves patient outcomes [15]. Organizations that cultivate open communication channels experience fewer adverse events, underscoring the necessity of a supportive communication framework [16]. Additionally, Social Exchange Theory suggests that employees weigh the costs and benefits of voicing their concerns. If they perceive the risks (e.g., retaliation, lack of support) to outweigh the benefits, they are more likely to remain silent [17].

Previous studies have explored the impact of communication and feedback systems on patient safety and organizational effectiveness. Research indicates that healthcare professionals often feel inhibited from voicing concerns due to fear of retaliation or lack of support from management [10, 18]. This aligns with findings suggesting that organizational culture is pivotal in encouraging or discouraging open dialogue among team members [7].

Various questionnaires have been developed to assess this concept in organizational silence. While tools like the Organizational Silence Scale by Van Dyne et al. [19] and the Organizational Silence Inventory by Knoll and Van Dick [20] offer valuable insights, they lack specificity regarding the unique challenges faced by healthcare professionals in high-stakes environments. One significant limitation of these instruments is their general applicability, which often fails to account for the specific dynamics and pressures within healthcare settings. For example, healthcare professionals frequently encounter situations where speaking up about patient safety issues is critical [21]. However, research indicated that many team members refrain from voicing their concerns until these issues escalate to critical levels. This delay in communication can severely compromise patient safety and care quality, leading to adverse outcomes [22].

Moreover, the existing tools do not adequately address the psychological barriers that healthcare professionals face. Factors such as fear of retaliation—where individuals worry about negative repercussions from colleagues or management—play a crucial role in fostering silence [23, 24]. Additionally, inadequate support from management can exacerbate feelings of isolation and disempowerment among staff, making them less likely to speak up. The existing questionnaires often overlook these emotional and contextual factors, resulting in a lack of actionable insights for improving team communication [21, 24].

In Turkey, a scale was developed by Yalçın and Baykal to measure organizational silence behavior among nurses to minimize the gap in the literature review. The reassessment of the organizational silence behavior scale (OSBS) for healthcare professionals is crucial due to the evolving nature of healthcare systems and the increasing recognition of the impact of organizational silence on patient outcomes. Unlike existing scales, the OSBS is specifically tailored to reflect the dynamics of healthcare settings, incorporating factors such as hierarchical structures and interdisciplinary communication challenges prevalent in clinical environments [21].

The importance of developing a culturally appropriate tool for measuring organizational silence in the Iranian context cannot be overstated. A tailored instrument, such as the OSBS-P, is necessary to accurately reflect the organizational dynamics and communication challenges faced by healthcare professionals in Iran. This study aims to fill the research gap by providing a psychometrically validated scale that captures the nuances of organizational silence behaviors specific to Iranian clinical settings.

This research contributes to the existing body of knowledge by offering new insights into the factors influencing organizational silence among clinical nurses in Iran. It emphasizes the need for a new tool with valid and reliable psychometric properties, addressing the limitations of existing scales.

### Statement of the problem

It is crucial to investigate the phenomenon of organizational silence in Iranian healthcare environments [25]. Organizational silence, where employees are hesitant to address critical issues, is a significant concern in healthcare settings due to its potential adverse impact on patient safety and care quality [10]. By delving into the causes and manifestations of organizational silence, the research aimed to gain insights that can guide targeted interventions to mitigate its effects and promote a culture of open communication and transparency within healthcare organizations. Ultimately, the findings of this research have the potential to improve healthcare outcomes by illuminating the dynamics of organizational silence and facilitating the implementation of interventions to foster open communication and employee voice, thereby enhancing patient safety and care quality in Iranian healthcare settings. The primary goal of this study was to translate and evaluate the psychometric properties of the Persian version of the Organizational Silence Behavior Scale (OSBS-P) among clinical nurses.

## Materials and methods

### Design and setting

From October 2023 to February 2024, we conducted a methodological study to assess the reliability and validity of the Persian version of the Organizational Silence Behavior Scale (OSBS-P) among clinical nurses. This validation process was a significant aspect of our research. We used convenience sampling to gather participants efficiently within our limited research timeframe. This approach allowed us to recruit participants from five educational-therapeutic hospitals in Ardabil province, Northwestern Iran.

Given our time and resource constraints, convenience sampling proved especially advantageous and enabled us to collect data from readily available and willing clinical nurses. To further clarify our methodological design, we referenced previous studies that employed convenience sampling in psychological research, underlining its effectiveness in similar contexts. For example, studies by Yalçın and Baykal [21] and Chung and Hwang [23] successfully utilized convenience sampling to investigate related phenomena, affirming the reliability of findings obtained through this method. Convenience sampling can yield valuable insights, especially in exploratory research or when access to a larger population is limited. However, it may also introduce selection bias and restrict the generalizability of the findings. Therefore, while we reference these studies to advocate for convenience sampling in our research context, we must consider its limitations and interpret the results cautiously. This balanced perspective fosters a deeper understanding of the implications of employing convenience sampling in our study [26].

### Participants

A survey was conducted on 338 clinical nurses who work in educational-therapeutic hospitals of Ardabil University of Medical Sciences in Iran. To be eligible for the study, participants had to voluntarily agree and possess at least a bachelor’s degree in nursing or a higher qualification. Additionally, they needed to have a minimum of six months of work experience in the field. The exclusion criterion for disqualification from the research was unanswered questions in the questionnaire.

### Instrument

The tool comprised two sections. The initial section consisted of a questionnaire to collect demographic information, including age, gender, work experience, and other relevant details. The second part of the questionnaire was the Organizational Silence Behavior Scale (OSBS-P), designed by Yalçın and Baykal [21] and administered to clinical nurses. The OSBS-P is a scale that comprises 32 items, which are further divided into four subscales. These subscales include silence climate (5 items), silence based on fear (12 items), acquiescence silence (10 items), and silence based on protecting the organization (5 items). The items in the scale are rated on a 5-point Likert scale ranging from 1 (never stay silent) to 5 (always stay silent). As the total score on the scale increases, the level of silence also increases (S1 File).

The Content Validity Index (CVI) for the original scale was measured at 0.86, indicating an adequate level of content validity. Before performing Confirmatory Factor Analysis (CFA), Exploratory Factor Analysis (EFA) was conducted to determine the suitability of the dataset for CFA. The KMO index was 0.945, and the results of Bartlett’s test showed χ^2^ = 14903.485; *p* < 0.001 (*n* = 485), indicating that the data were appropriate for factor analysis. The CFA results revealed a CMIN/DF of 3.54, RMSEA of 0.07, GFI of 0.92, CFI of 0.92, and NFI of 0.90, demonstrating a good fit for the model. Additionally, the Cronbach’s alpha for the overall scale was 0.93. Specifically, the reliability coefficients for the subscales were as follows: ’Silence based on fear’ (0.93), ’Silence based on protecting the organization’ (0.85), ’Silence climate’ (0.93), and ’Acquiescent silence’ (0.91).

### Psychometric evaluation

#### Translation procedure

The researchers sought permission from Dr. Begüm Yalçın, the developer of OSBS, to use the tool with Iranian clinical nurses. They followed the forward-backward method recommended by the WHO guidelines to translate the tool from English to Persian and back to ensure accuracy [27]. Two professional translators worked independently to translate the tool from English to Persian, which was evaluated and discussed by an expert panel comprising two nursing professors and two translators. The Persian version was finalized and subsequently back-translated into English by a translator without previous knowledge of the original version or the study’s objectives. The expert panel reviewed and refined this back-translated version against the original version (S2 File) to create the final Persian tool.

#### Face validity

A scale’s face validity was assessed using quantitative and qualitative approaches. Ten clinical nurses from different units were interviewed to evaluate the scale’s face validity. The nurses were asked to provide feedback on the items’ difficulty level, relevance to the measured concept, and presence of ambiguous language. The researchers recorded and reviewed the participants’ comments. Based on the feedback, any required modifications to the scale were made.

A structured approach was employed to assess the quantitative face validity of the scale. Clinical nurses were asked to evaluate the significance of each item on a five-point scale ranging from "unimportant" (1) to "important" (5). The impact score for each item was calculated by multiplying the percentage of respondents who rated the item as important (scores of 4 or 5) by the average importance rating assigned to that item. An item was considered significant if it had an impact score greater than 1.5. This method ensured that only items deemed relevant and meaningful by the target population of clinical nurses were included in the final scale [28].

#### Content validity

The content validity of the OSBS-P was evaluated using qualitative and quantitative methods. For the qualitative method, we conducted purposive sampling and administered the OSBS-P to ten experts, specifically nursing faculty members with diverse backgrounds. These faculty members were selected based on their expertise in various areas of nursing, including clinical practice, nursing education, and research. Their collective experience ensured a comprehensive evaluation of the scale. Following the qualitative examination, the experts provided feedback on several aspects of the OSBS-P, including grammar, terminology, and overall relevance to clinical nursing practice. This feedback was instrumental in refining the scale to meet the target population’s needs better.

To evaluate the precision of our scale, we conducted an assessment of its quantitative content validity. This was done using the content validity ratio (CVR) and the content validity index (CVI). A 3-point Likert scale was used to rate each item to determine the CVR. Experts rated each item as "essential," "useful but not essential," or "not essential" for the scale. The CVR formula was utilized to calculate the CVR. The formula considers the total number of invited experts (N) and the number of experts who ranked the item (Ne). The resulting ratio is CVR = (Ne—N/2) / (N/2). According to Lawshe’s table, a CVR value of 0.62 or higher is acceptable. Also, the items were rated by experts on a 4-point Likert scale to evaluate their relevance, clarity, and simplicity. The scale ranged from 1 for "not relevant" to 4 for "completely relevant." Items with CVIs greater than 0.79 were deemed acceptable [29].

To ensure an accurate assessment, an examination was conducted to detect any floor and ceiling effects that could suggest content validity issues. A floor effect occurs when over 15% of participants attain the lowest possible score on a particular tool, indicating that the tool lacks items representing the minimum intensity of the phenomenon. Conversely, a ceiling effect occurs when more than 15% of participants achieve the highest possible score on the tool, suggesting a lack of items representing the maximum intensity of the phenomenon. If these effects exceed 15%, the tool may be considered to have inadequate content validity [30].

#### Construct validity

The scale OSBS-P was created by utilizing exploratory factor analysis (EFA). In contrast, this research employed confirmatory factor analysis (CFA) to assess the construct validity [31]. Several methods are available to determine the construct validity of a measurement instrument, such as Confirmatory Factor Analysis (CFA) and Exploratory Factor Analysis (EFA). However, research suggests that CFA is generally considered more effective than EFA. CFA allows researchers to test a pre-specified theoretical model of the underlying constructs. At the same time, EFA involves an exploratory approach that may not necessarily align with the theoretical model. Therefore, CFA is often preferred when the goal is to confirm a specific hypothesis or construct [32]. The current study estimated the parameters using Maximum Likelihood Estimation (MLE). The model fit indices were evaluated based on the following criteria: χ^2^/df ratio less than 3, RMSEA less than 0.08, GFI greater than 0.90, CFI greater than 0.90, TLI greater than 0.90, NFI greater than 0.90, and AGFI greater than 0.80 [33]. Statistically significant factor loadings were those above 0.3 and T-values above 1.96, as per Hair et al. [34].

There are varying opinions on what constitutes an adequate sample size for a study. Some experts recommend a minimum of 100 participants, while others suggest a larger sample size of 400 or more. The number of participants needed may also depend on other factors, such as the complexity and number of items in the measuring tool [34–40]. In our study, we employed the OSBS-P, which comprised 32 items. Adhering to the general guideline of having 5–10 participants for each item [41], we recruited 12 participants per item, culminating in 384 clinical nurses through convenience sampling. Notably, 88% of these participants completed the survey. After carefully analyzing the valid responses, we selected 338 participants for the CFA. This sample size was adequate to ensure a robust structural validity analysis while accurately representing the target population and minimizing the risk of bias or error.

#### Convergent and discriminant validity

Hair et al. defined convergent validity as the degree to which a construct is characterized by its items’ similarity and high common variance. In contrast, divergent validity is demonstrated when a construct’s items are dissimilar and unrelated [34]. To evaluate the accuracy and consistency of the OSBS-P scale, we used the Fornell and Larcker criterion, along with key parameters such as Composite Reliability (CR), Average Variance Extracted (AVE), Maximum Shared Squared Variance (MSV), and Average Shared Squared Variance (ASV) [42, 43]. To establish convergent validity, we looked for AVE values greater than 0.5 and CR values greater than 0.7, with CR being greater than AVE [44]. It should be noted that if the AVE exceeds both the MSV and the ASV, it is a sign that the measurements employed in the analysis possess reliable discriminant validity [34]. To confirm discriminant validity, the researchers utilized the Heterotrait-Monotrait (HTMT) correlation ratio criterion developed by Henseler et al. [45]. As per Henseler et al.’s recommendation, all values in the HTMT matrix must be less than 0.9 [45].

#### Reliability

We assessed the internal consistency of the instrument and its dimensions using Cronbach’s alpha coefficient (α), McDonald’s omega (ω), Coefficient H, and mean inter-item correlation (ρ) to confirm that the items within each scale measure the same construct reliably. For the results to be acceptable, the values of α, ω, and H should be greater than 0.7 [46, 47]. Furthermore, Coefficient H demonstrated the highest level of reliability among the subscales [46–48]. It’s worth noting that internal consistency can be measured regardless of the number of items or sample size since mean inter-item correlation remains independent [43, 49, 50]. An inter-item correlation ranging from 0.15 to 0.5 is commonly considered optimal. If the correlation falls below 0.15, it may suggest a weak relationship between the items. Conversely, the correlation above 0.5 may indicate redundancy among the items [51].

The Intraclass Correlation Coefficient (ICC) was used to determine the stability of the OSBS-P. The data were collected from 40 clinical nurses by simple random sampling over two weeks to assess test-retest reliability. The acceptable threshold was an ICC value of 0.75 or above [52].

### Data analysis

Two methods were used to detect outliers in the data. The first method involved examining the skewness and kurtosis values for univariate outliers. Values larger than ±3 and ±8, respectively, were considered outliers. The second approach employed the Mahalanobis squared distance technique to identify multivariate outliers. The approach sets a statistical significance threshold at *p* < 0.001, indicating that only outliers with a very low probability of occurring by chance will be identified. The suitability of a normal distribution for multivariate variables was assessed using Mardia’s coefficient. If the computed Mardia coefficient was above 8, it was determined that the data did not adhere to a normal distribution [53].

The study employed IBM SPSS Statistics for Windows, version 24.0 (IBM Corp., Armonk, NY, USA) for performing descriptive statistical analysis (such as mean, standard deviation (SD), number, and percentage). The CFA and structured equation modeling (SEM) were also conducted using IBM SPSS AMOS, version 24.0. The predetermined statistical significance level was established at *p* < 0.05.

### Ethical considerations

The Research Ethics Committee of Ardabil University of Medical Sciences, Iran, has approved the research proposal with the ethics code (IR.ARUMS.REC.1402.062). The research project followed the principles outlined in the Declaration of Helsinki. Before the commencement of the study, written informed consent was acquired from all participating individuals. Detailed information regarding the study’s objectives, methods, nature, and duration was provided to each participant thoroughly. This included a comprehensive explanation of the potential benefits, risks, and any potential discomforts that may be experienced. Participants were also informed of their right to withdraw from the study at any time.

## Results

### Participant characteristics

The demographic characteristics of the participants in our study, which included 338 individuals, revealed a mean age of 35.75 years (SD = 4.33) and an average working experience of 9.52 years (SD = 4.25). The sample comprised 39.1% male (*n* = 132) and 60.9% female (*n* = 206) participants. Regarding marital status, 41.7% of the participants were single (*n* = 141), while 58.3% were married (*n* = 197). Education-wise, the majority held a bachelor’s degree (71.6%, *n* = 242), with 28.4% possessing a master’s degree (*n* = 96). Participants were also divided based on their working departments, with 36.4% in medical (*n* = 123), 20.1% in surgical (*n* = 68), 9.5% in emergency (*n* = 32), 9.5% in ICU (*n* = 32), 6.8% in pediatric (*n* = 23), and 17.7% in other departments (*n* = 60).

### Face validity

During the face validity process, we received feedback from clinical nurses regarding the translated version of the OSBS-P scale. The nurses highlighted several key areas for improvement: some items were a little complex, prompting suggestions to simplify the language for better clarity. Instances of ambiguous language were identified, and the nurses recommended rephrasing these items to ensure consistent interpretation among respondents. The researchers recorded and reviewed the participants’ comments. Based on this feedback, any required modifications to the scale were made, enhancing its clarity and relevance to clinical practice. As a result, all items in the translated version received a score of 1.5 or higher, ranging from 2.5 to 3.8 (**Table 1**). Accordingly, we discovered that all the items present in the original OSBS-P scale were deemed necessary by the participants, and none of them were removed during the face validity process. Keeping these findings in mind, we retained all the items to proceed with further stages of our study.

**Table 1:** The results for the face and content validity of the OSBS-P (*n* = 338).

Item	Impact score	CVI			CVR
		Relevance	Clarity	Simplicity	
1	2.5	1	1	1	1
2	2.7	1	1	1	1
3	3.8	0.9	1	1	0.8
4	2.5	1	1	0.9	0.9
5	3.2	1	1	1	0.9
6	3.7	1	0.8	1	1
7	3.8	0.9	1	1	1
8	2.5	1	1	0.9	1
9	3.4	1	1	1	0.8
10	2.7	0.9	1	1	0.7
11	2.7	1	1	1	0.7
12	2.5	1	0.9	1	0.8
13	3.4	0.8	0.9	0.9	0.7
14	3.7	1	1	0.9	1
15	2.7	1	1	1	0.9
16	3.8	1	1	1	0.7
17	3.7	1	1	1	1
18	3.7	1	1	1	1
19	2.5	1	1	0.8	1
20	3.4	1	1	1	1
21	2.7	0.8	1	1	0.9
22	3.7	1	1	1	0.9
23	2.5	1	0.9	1	0.9
24	3.4	0.8	0.9	0.9	0.8
25	3.4	1	1	0.9	0.7
26	2.7	1	1	1	1
27	3.8	1	1	1	0.9
28	3.7	1	1	1	0.7
29	3.7	1	1	0.9	1
30	2.7	1	1	1	1
31	3.8	0.9	1	1	1
32	3.7	1	1	0.9	1

### Content validity

According to the results, each item had a CVR value above 0.62, varying from 0.7 to 1. The Waltz and Bausell method yielded a CVI of over 0.79 for each item (ranging from 0.8 to 1) (**Table 1**). During the qualitative phase of the study, the experts found that the 5-point Likert scale ranging from 1 (never stay silent) to 5 (always stay silent) had an appropriate ranking scale that effectively measured the intended constructs. Additionally, they concluded that the grammar, wording, item allocation, and scaling of the response category were all suitable for the targeted population and research objectives. This suggests that the response category used in the survey was well-designed and reliable for collecting valuable data.

Moreover, no floor or ceiling effects were observed when most participants scored at the lowest or highest possible level, respectively. Accordingly, the percentage of participants who scored at the lowest or highest possible level was less than 15% for all measures. This indicates that the OSBS-P is a reliable and valid tool for measuring the constructs it was designed to assess (**Table 2**).

**Table 2:** Descriptive statistics, floor and ceiling effects of the 32-item OSBS-P (*n* = 338).

Dimensions	No. of item	Possible range	Mean ± SD	Skewness	Kurtosis	Floor effect (%)	Ceiling effect (%)
Silence climate	5	1–5	3.61 ± 0.89	-0.479	-1.023	4 (1.2%)	21 (6.2%)
Silence based on fear	12	1–5	3.02 ± 0.68	-0.506	-1.015	2 (0.6%)	26 (7.7%)
Acquiescence silence	5	1–5	3.12 ± 0.67	-0.463	-0.889	10 (3.0%)	23 (6.8%)
Silence based on protecting the organization	10	1–5	3. 48 ± 0.85	-0.312	-0.959	7 (2.1%)	37 (10.9%)
Total (32-item OSBS-P)	32	1–5	3.11 ± 0.47	-0.484	-1.110	2 (0.6%)	3 (0.9%)

### Descriptive statistics of the 32-item OSBS-P

The scale’s overall mean score was 3.11 (0.47). The mean scores for the subscales of silence climate, silence based on fear, acquiescence silence, and silence based on protecting the organization were 3.61 (0.89), 3.02 (0.68), 3.12 (0.67), and 3.48 (0.85), respectively. According to the findings, the study’s results showed that the overall scale and subscales had negative skewness. The negative skewness means that most participants had favorable attitudes towards the items in the scale, which could suggest a high degree of satisfaction or agreement with the statements presented. This information provides valuable insight into the perceptions and attitudes of the participants towards the topic under investigation (**Table 2**).

### Construct validity

**Fig 1:**
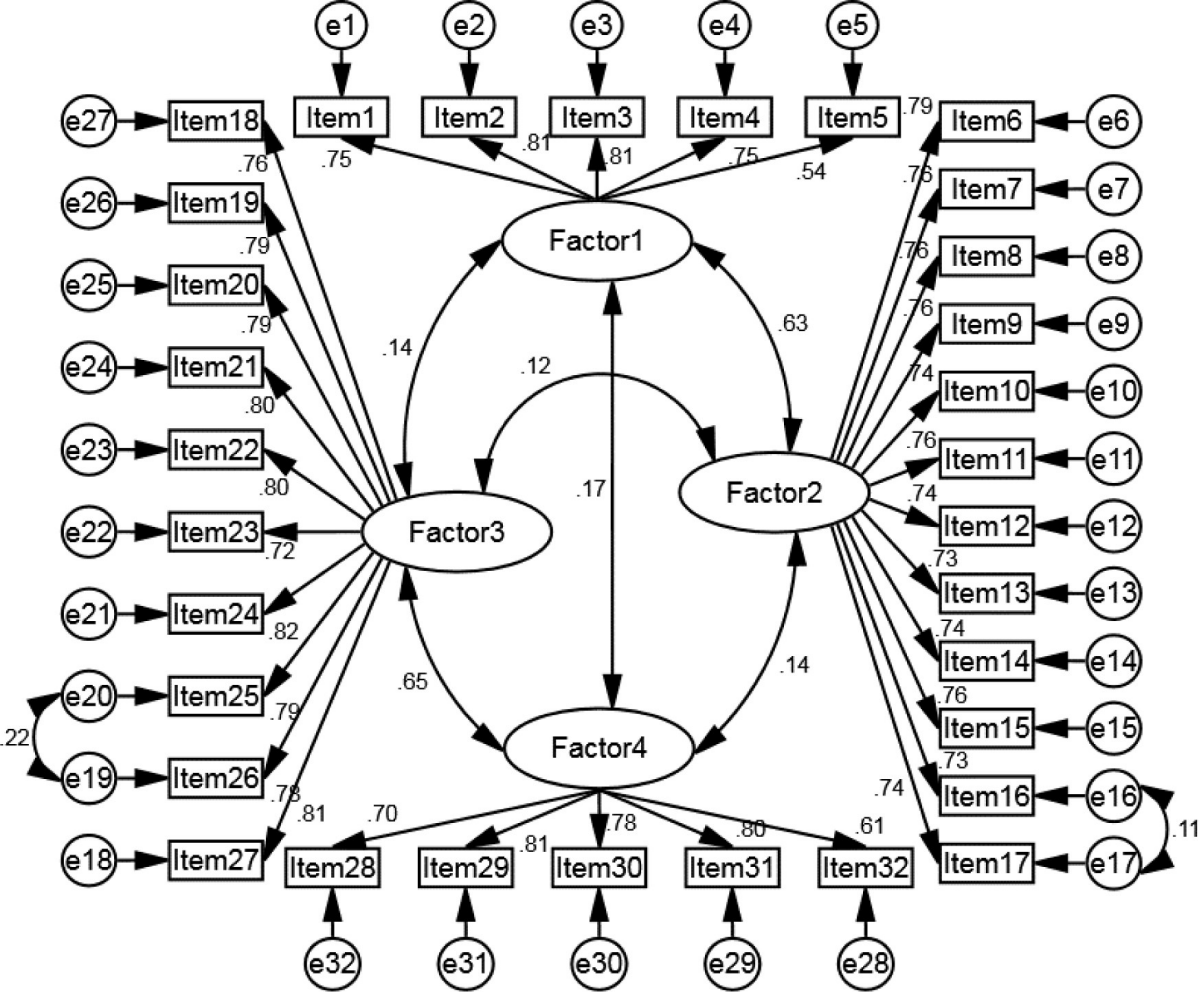
Confirmatory factor analysis and the standardized parameter estimates of the modified model of the OSBS-P scale (*n* = 338).

**Fig 1** shows the OSBS-P structure, where the latent factors 1 to 4 are silence climate, silence based on fear, acquiescence silence, and silence based on protecting the organization, respectively. The CFA findings conducted using a four-factor model revealed that all the items in the model had a factor loading of 0.3 or higher, ranging from 0.54 to 0.81. This suggests that none of the items needed to be eliminated from the model, and the results were statistically significant with a *p* < 0.001. The statistical analysis using T-value tests revealed a significant relationship between all latent factors and their corresponding items. It is worth mentioning that the T-value for all items was greater than 1.96, indicating a strong association between the factors and their observed variables. The proposed model of OSBS-P and its concepts are deemed acceptable overall based on the goodness-of-fit indices: χ^2^ = 1254.65, df = 456, *p* < 0.001, χ^2^/df = 2.751, RMSEA = 0.052, GFI = 0.913, CFI = 0.938, TLI = 0.933, NFI = 0.939, and AGFI = 0.877 (**Table 3**). It was determined that four latent factors approved this structure.

**Table 3:** The goodness of fit statistics for CFA models of the OSBS-P (*n* = 338).

Indices	χ^2^	df	*p-*value	χ^2^/df	RMSEA	GFI	CFI	TLI	NFI	AGFI
CFA model	1213.37	571	0.001	2.125	0.055	0.919	0.927	0.921	0.927	0.863
Acceptable values	-	-	> 0.05	< 3	< 0.08	> 0.90	> 0.90	> 0.90	> 0.90	> 0.80

Abbreviations: χ^2^/df, Ratio of chi-square to its degree of freedom; RMSEA, Root Mean Square Error of Approximation; GFI, Goodness of Fit Index; CFI, Comparative Fit Index; TLI, Tucker-Lewis index; NFI, Incremental Fit Index; AGFI, Adjusted Goodness of Fit Index.

### Convergent and discriminant validity

The results presented in **Table 4** indicate that the latent factors under study exhibit strong convergent validity. This is evidenced by their high CR and AVE values, surpassing the recommended thresholds of 0.7 and 0.5, respectively, with CR being greater than AVE. These findings suggest that the measurement model used to assess the latent constructs is reliable and valid, providing robust support for the study’s theoretical framework. The discriminant validity of the latent factors is confirmed by the MSV and ASV values being lower than AVE. The HTMT matrix analysis also reveals that all values are less than 0.9, as presented in **Table 4**.

**Table 4:** Indices of the convergent and discriminant validity of the OSBS-P (*n* = 338).

Dimensions	CR	AVE	MSV	ASV	HTMT			
					silence climate	silence based on fear	acquiescence silence	silence based on protecting the organization
Silence climate	0.855	0.545	0.396	0.148	-			
Silence based on fear	0.939	0.564	0.396	0.143	0.411	-		
Acquiescence silence	0.941	0.618	0.422	0.152	0.243	0.386	-	
Silence based on protecting the organization	0.864	0.681	0.422	0.157	0.339	0.413	0.694	-

Abbreviations: CR, Composite Reliability; AVE, Average Variance Extracted; MSV, Maximum Shared Squared Variance; ASV, Average Shared Squared Variance; HTMT, Heterotrait-Monotrait ratio of correlation.

### Reliability

The internal consistency of the scale was assessed using various statistical measures such as alpha coefficient (α = 0.969), MacDonald’s omega coefficient (ω = 0.971), coefficient H (H = 0.977), and mean inter-item correlation (ρ = 0.494). These measures indicate that the OSBS-P scale is a reliable and consistent tool for assessing the construct it aims to measure. Also, MacDonald’s omega coefficient and H coefficient values for four factors (subscales) were above 0.7. On the other hand, mean inter-item correlation values for all factors were between 0.15 and 0.5, which indicates excellent and acceptable internal consistency (**Table 5**).

**Table 5:** Internal consistency and stability for the OSBS-P (*n* = 338).

Dimensions	α	ω	H	ρ	ICC (95% CI)
Silence climate	0.812	0.827	0.871	0.463	0.941 (0.904–0.961)
Silence based on fear	0.913	0.923	0.939	0.466	0.896 (0.790–0.921)
Acquiescence silence	0.904	0.917	0.935	0.485	0.901 (0.913–0.978)
Silence based on protecting the organization	0.817	0.823	0.871	0.471	0.898 (0.759–0.916)
Total (32-item OSBS-P)	0.969	0.971	0.977	0.494	0.951 (0.919–0.945)

Abbreviations: α, Cronbach’s alpha; ω, McDonald’s mega coefficient; H, Coefficient H; ρ, Mean inter-item correlation; ICC, Intraclass correlation coefficient; CI, Confidence interval.

The ICC was utilized to assess the OSBS-P’s stability across all dimensions. The overall ICC was 0.951. Notably, the ICC values for all dimensions were above 0.75, demonstrating a high level of agreement among the raters. These findings reinforce the OSBS-P’s reliability, instilling confidence in its use in future research and clinical settings (**Table 5**).

## Discussion

This study aimed to translate the Organizational Silence Behaviors Scale (OSBS) into Persian and evaluate its psychometric properties among clinical nurses working in educational-therapeutic hospitals of Ardabil, Iran. The study involved the participation of 338 clinical nurses selected through convenience sampling. The translated version of the OSBS was administered to the participants, and the data was analyzed using various statistical methods to assess the reliability and validity of the scale. This scale can be utilized as an effective instrument to measure and objectively represent the extent of organizational silence within the healthcare environment. Furthermore, this tool fulfills the requirement stated in prior studies that emphasized the importance of avoiding organizational silence, improving the working conditions for healthcare professionals, detecting trends in organizational silence, and encouraging healthcare professionals to communicate comfortably with their superiors and subordinates [54].

The face validity assessment indicated that clinical nurses found the translated OSBS-P items to be relevant and necessary, with scores ranging from 2.5 to 3.8. This valuable feedback prompted significant improvements in item clarity and language simplicity. In contrast, the original Turkish version [21] encountered challenges with item complexity, leading to the removal of nine items due to low correlation coefficients (r = 0.19 to 0.74). Our proactive approach in incorporating feedback from end-users not only enhanced the scale’s clarity but also ensured its resonance with the target population. This participatory method is crucial as it aligns with best practices in survey design, emphasizing the importance of user-centered approaches in developing valid measurement tools.

Validity is a concept that refers to the extent to which a measurement tool accurately measures what it is intended to measure in the real world. It measures how well the tool aligns with the natural world and accurately captures the phenomenon being studied [55]. The scale’s content validity results further confirmed its robustness, with all items having CVR values exceeding 0.62 and CVI values above 0.79. In contrast to the original scale [21], which underwent item reduction based on similar validity assessments and resulted in a final count of 32 items, our study retained all items. We enhanced their relevance and clarity, improving the scale’s applicability in clinical settings. Our data showed no floor or ceiling effects, indicating that the OSBS-P scale could effectively capture a wide range of responses and reinforce its reliability as a measurement tool.

The construct validity of a scale refers to how accurately and consistently it measures the concept or construct it is designed to measure. In simpler terms, it determines whether the assessment instrument is measuring what it is intended to measure and whether the results obtained from the instrument reflect the underlying construct being measured [56]. The results of the CFA confirmed the statistical robustness of our four-factor model, with all items showing significant factor loadings (≥ 0.3). This represented a considerable improvement over the original scale, where several items had to be removed due to insufficient factor loadings. Our CFA revealed strong connections between the latent factors and their respective items, providing further support for the theoretical framework of the OSBS-P. The goodness-of-fit indices indicated that our model was acceptable, underscoring the effectiveness of our adaptation process. The original version’s use of EFA before CFA had resulted in a somewhat unstable model, requiring the removal of multiple items to attain satisfactory fit indices [21]. Our study’s methodology, incorporating qualitative and quantitative validation techniques, produced a more dependable and valid representation of the construct.

Based on the results of a reliability analysis, it has been determined that for a scale to be considered reliable, it must consistently reflect the underlying structure it intends to measure. This means that the scale should produce consistent and reliable results over time and accurately assess the construct it is designed to measure [57]. The internal consistency of the OSBS-P was evaluated using various statistical measures, resulting in a Cronbach’s alpha of 0.969, signifying excellent reliability. This surpasses the original Turkish version [21], which reported a Cronbach’s alpha of 0.93. The strong intraclass correlation coefficient (ICC = 0.951) further reinforces the scale’s stability across dimensions. These results indicate that our adapted version of the OSBS-P maintains high reliability and enhances the original tool’s consistency. The rigorous methodology we employed in assessing internal consistency, including the calculation of MacDonald’s omega coefficient, coefficient H, and mean inter-item correlations, establishes a strong basis for the reliability of our findings.

Our study showed that the translated OSBS-P is a valid and reliable tool for evaluating organizational silence in clinical settings. By overcoming previous research limitations and integrating expert input into our validation process, we have developed a strong instrument to improve comprehension of communication barriers within healthcare organizations and to facilitate future studies on their influence on clinical practice.

The authors have identified several limitations in evaluating the validity and reliability of the OSBS-P. The process of conducting CFA involved selecting participants using a convenience sampling method and recruiting individuals from educational and therapeutic centers affiliated with Ardabil University of Medical Sciences. While this approach facilitated efficient participant recruitment, it’s crucial to acknowledge that the external validity of the findings may be limited. Convenience sampling may not accurately represent the broader population, potentially weakening the generalizability of the results. It’s important to always consider this limitation when interpreting the findings of the CFA. Additionally, while the study included 338 clinical nurses, surpassing the minimum sample size requirement, it is crucial to recognize that the sample’s demographic characteristics may not reflect the diversity of clinical nurses in other regions or countries. Factors such as age, gender, years of experience, and cultural background could influence the psychometric properties of the OSBS-P, and the lack of representation from various demographics may limit the applicability of the findings. Another limitation is the reliance on self-reported measures, which can introduce biases such as social desirability bias, where participants may respond in a manner they believe is favorable rather than providing honest answers. This could affect the accuracy of the data collected and, consequently, the results of the CFA. Lastly, the study’s limited data collection period does not allow for examining changes over time or the causal relationships between the variables. Longitudinal studies would be beneficial in understanding how the psychometric properties of the OSBS-P may evolve in different settings or populations.

Despite the limitations, the study presented several notable strengths that contributed to understanding the OSBS-P’s psychometric properties. First and foremost, the study successfully conducted CFA on a substantial sample of 338 clinical nurses, which exceeded the minimum sample size requirements for robust statistical analysis. This large sample enhanced the reliability of the findings and provided a solid foundation for evaluating the scale’s psychometric characteristics. Furthermore, the study’s methodological rigor in employing CFA reflected a commitment to ensuring the OSBS-P’s psychometric soundness. By utilizing established statistical techniques, the authors provided a comprehensive scale evaluation, adding credibility to the results and reinforcing psychometric assessment’s importance in instrument development.

## Conclusions

The Persian version of the Organizational Silence Behavior Scale (OSBS-P) demonstrates sound psychometric properties, including validity, reliability, and internal consistency. Due to its ease of administration, this tool can assess organizational silence behavior among clinical nurses. Therefore, future research should utilize larger sample sizes and include various hospital departments and groups to improve OSBS-P among clinical nurses in the healthcare environment. Thus, the study that has been conducted can prove to be extremely beneficial for nursing managers. It aims to identify and analyze the factors contributing to organizational silence behavior among clinical nurses. This analysis is based on using OSBS’ dimensions, which can help identify the strengths and weaknesses of the clinical nursing staff. By examining these factors, healthcare system managers and nursing policymakers can take necessary steps to improve the workplace environment and ensure clinical nurses can perform their duties effectively and efficiently. With this study, it is hoped that the issues related to organizational silence behavior among clinical nurses can be addressed and resolved, ultimately leading to better patient care and outcomes.

## Supporting information

S1 File Persian version of the OSBS. (DOCX)

S2 File Original scale (Turkish). (DOCX)
