# Supplementary material for: Psychometric evaluation and translation of the Persian version of the Organizational Silence Behavior Scale (OSBS-P) for clinical nurses
Source: PLoS One. 2024 Dec 30;19(12):e0314155. doi: 10.1371/journal.pone.0314155 (PMC11684606; doi:10.1371/journal.pone.0314155)
Supplement: S1 File — (DOCX) [file pone.0314155.s001.docx]

**Original version of the Organizational Silence Behavior Scale (OSBS)**

|  |  | **Never stay**  **silent** | **Stay rarely**  **silent** | **Sometimes**  **stay silent** | **Occasional**  **ly stay**  **silent** | **Always**  **stay silent** |
| --- | --- | --- | --- | --- | --- | --- |
| **Silence**  **Climate** | If the employees tend to relate well to with their managers |  |  |  |  |  |
|  | If my manager is not acting empathetically to the issues |  |  |  |  |  |
|  | If I don't find my manager dependable |  |  |  |  |  |
|  | If my administrators are not open to feedback |  |  |  |  |  |
|  | If I'm afraid of being excluded from the team |  |  |  |  |  |
| **Silence Based On Fear** | If I am obligated to look after my family |  |  |  |  |  |
|  | If I'm afraid of getting a low performance evaluation |  |  |  |  |  |
|  | If I'm afraid of being punished |  |  |  |  |  |
|  | If I think that I will be exposed to mobbing by my manager |  |  |  |  |  |
|  | If I'm afraid of opposing my manager's opinion |  |  |  |  |  |
|  | If I'm afraid of to be fired |  |  |  |  |  |
|  | If I have the possibility of exposing to violence |  |  |  |  |  |
|  | If I'm afraid of excessive reactions |  |  |  |  |  |
|  | If I think I might be harmed by saying my opinion |  |  |  |  |  |
|  | If I think that I may have problems in collaboration in the workplace |  |  |  |  |  |
|  | If I'm afraid of misunderstanding |  |  |  |  |  |
|  | If I'm afraid of being unsuccessful |  |  |  |  |  |
| **Acquiesce Silence** | If I think staying silent is the solution |  |  |  |  |  |
|  | If I'm tired of not seeing the results of my efforts |  |  |  |  |  |
|  | If I can't get feedback about my suggestions |  |  |  |  |  |
|  | If I don't believe there will be a change |  |  |  |  |  |
|  | If I don't want to be a part of the institution |  |  |  |  |  |
|  | If I think there's no need to talk or raise my concerns |  |  |  |  |  |
|  | If I'm starting to not care about my organization |  |  |  |  |  |
|  | If I'm tired of dealing with my co-workers |  |  |  |  |  |
|  | If my commitment to the institution is reduced |  |  |  |  |  |
|  | If I think that I am being devalued |  |  |  |  |  |
| **Silence Based on**  **Protecting the**  **Organization** | If I think my incompetency will harm my organization |  |  |  |  |  |
|  | If there is a lack of technological infrastructure and equipment in my organization |  |  |  |  |  |
|  | If I think that my organization’s competitiveness will decrease |  |  |  |  |  |
|  | If I think, it would harm the reputation of my organization. |  |  |  |  |  |
|  | If I have confidential information about the my organization’s new investments |  |  |  |  |  |

**بسمه تعالی**

مشخصات دموگرافیک

**سن: ......... جنسیت:** مرد🞎 زن🞎

**وضعیت تاهل:** مجرد🞎 متاهل🞎

**سطح تحصیلات:** کارشناسی🞎 کارشناسی ارشد🞎

**بخش محل کار:** داخلی🞎 جراحی🞎 اورژانس🞎 🞎ICU کودکان🞎 اتاق عمل🞎 سایر🞎

**سابقه کاری: ...........**

**پرسشنامه سکوت سازمانی**

| گویه ها | هرگز سکوت نمی کنم | به ندرت سکوت می کنم | گاها سکوت می کنم | معمولا سکوت می کنم | همیشه سکوت می کنم |
| --- | --- | --- | --- | --- | --- |
| 1. اگر کارکنان تمایل داشته باشند با مدیران خود روابط خوبی برقرار کنند. |  |  |  |  |  |
| 2. اگر مدیر من با همدلی به مسائل نپردازد. |  |  |  |  |  |
| 3. اگر مدیرم را قابل اعتماد ندانم. |  |  |  |  |  |
| 4. اگر مدیران من آماده بازخورد نباشند. |  |  |  |  |  |
| 5. اگر از حذف شدن از تیم بترسم. |  |  |  |  |  |
| 6. اگر مسئولیت مراقبت از شخصی را داشته باشم. |  |  |  |  |  |
| 7. اگر از ارزیابی ضعیف عملکردم تردید داشته باشم. |  |  |  |  |  |
| 8. اگر از تنبیه شدن بترسم. |  |  |  |  |  |
| 9. اگر قرار باشد از جانب مدیرم کوچک شمرده شوم. |  |  |  |  |  |
| 10. اگر از مخالفت با نظر مدیرم بترسم. |  |  |  |  |  |
| 11. اگر از اخراج شدن بترسم. |  |  |  |  |  |
| 12. اگر احتمال قرار گرفتنم در معرض خشونت یا آسیب فیزیکی باشد. |  |  |  |  |  |
| 13. اگر از نمایش رفتار بیش از حد واکنشی تردید داشته باشم. |  |  |  |  |  |
| 14. اگر فکر کنم ممکن است با گفتن نظرم آسیب ببینم. |  |  |  |  |  |
| 15. اگر قرار باشد در محیط کارم در کار تیمی با مشکل مواجه شوم. |  |  |  |  |  |
| 16. اگر از سوءتفاهم بترسم. |  |  |  |  |  |
| 17. اگر از شکست خوردن بترسم. |  |  |  |  |  |
| 18. اگر فکر کنم ساکت ماندن راه حل است. |  |  |  |  |  |
| 19. اگر از ندیدن نتیجه تلاشم خسته شده باشم. |  |  |  |  |  |
| 20. اگر نتوانم درباره پیشنهاداتم بازخورد دریافت کنم. |  |  |  |  |  |
| 21. اگر باور نکنم که تغییر اتفاق خواهد افتاد. |  |  |  |  |  |
| 22. اگر نخواهم بخشی از موسسه (نهاد) باشم. |  |  |  |  |  |
| 23. اگر فکر کنم نیازی به صحبت کردن وجود ندارد. |  |  |  |  |  |
| 24. اگر من شروع به بی توجهی به سازمانم کنم. |  |  |  |  |  |
| 25. اگر از جر و بحث با انسان ها خسته شده باشم. |  |  |  |  |  |
| 26. اگر تعهدم به سازمان کم شده باشد. |  |  |  |  |  |
| 27. اگر فکر کنم بی ارزش شده ام. |  |  |  |  |  |
| 28. اگر فکر کنم بی کفایتی من به سازمانم آسیب می رساند. |  |  |  |  |  |
| 29. اگر زیرساخت ها و تجهیزات فناورانه در سازمان من کم باشد. |  |  |  |  |  |
| 30. اگر فکر کنم که رقابت پذیری سازمانم کاهش یافته است. |  |  |  |  |  |
| 31. اگر فکر کنم به اعتبار سازمانم لطمه خواهد خورد. |  |  |  |  |  |
| 32. اگر اطلاعات محرمانه ای در مورد سرمایه گذاری های جدید سازمانم داشته باشم. |  |  |  |  |  |
