# Supplementary material for: Psychometric evaluation and translation of the Persian version of the Organizational Silence Behavior Scale (OSBS-P) for clinical nurses
Source: PLoS One. 2024 Dec 30;19(12):e0314155. doi: 10.1371/journal.pone.0314155 (PMC11684606; doi:10.1371/journal.pone.0314155)
Supplement: S2 File — (DOCX) [file pone.0314155.s002.docx]

|  |  | **Hiçbir zaman sessiz kalmam** | **Çok nadir sessiz kalırım** | **Bazen sessiz kalırım** | **Genellikle sessiz kalırım** | **Her zaman sessiz kalırım** |
| --- | --- | --- | --- | --- | --- | --- |
| **Sessizlik İklimi** | 1. Çalışanların yöneticileriyle iyi ilişki kurma eğilimi gösteriyorsa |  |  |  |  |  |
|  | 1. Yöneticim konuya empatiyle yaklaşmıyorsa |  |  |  |  |  |
|  | 1. Yöneticimi güvenilir bulmuyorsam |  |  |  |  |  |
|  | 1. Yöneticilerim geri bildirime açık değilse |  |  |  |  |  |
|  | 1. Ekip içinde dışlanmaktan korkuyorsam |  |  |  |  |  |
| **Korkuya dayalı sessizlik** | 1. Bakmakla yükümlü olduğum kişiler varsa |  |  |  |  |  |
|  | 1. Performansımın düşük değerlendirilmesinden çekiniyorsam |  |  |  |  |  |
|  | 1. Ceza sisteminin işletilmesinden korkuyorsam |  |  |  |  |  |
|  | 1. Yöneticim tarafından mobinge maruz bırakılacaksam |  |  |  |  |  |
|  | 1. Yöneticimin görüşüne karşı çıkmaktan korkuyorsam |  |  |  |  |  |
|  | 1. İşten atılma korkusu yaşıyorsam |  |  |  |  |  |
|  | 1. Şiddet/fiziksel zarar görme olasılığım varsa |  |  |  |  |  |
|  | 1. Aşırı tepkisel davranışlar gösterilmesinden çekiniyorsam |  |  |  |  |  |
|  | 1. Fikrimi söylemekten dolayı zarar göreceksem |  |  |  |  |  |
|  | 1. Çalışma ortamında işbirliğinde sorun yaşayacaksam |  |  |  |  |  |
|  | 1. Yanlış anlaşılmaktan korkuyorsam |  |  |  |  |  |
|  | 1. Başarısız olmaktan korkuyorsam |  |  |  |  |  |
| **Kabullenici Sessizlik** | 1. Sessiz almanın çözüm olduğunu düşünüyorsam |  |  |  |  |  |
|  | 1. Uğraşılarımın karşılığını almamaktan bıkmışsam |  |  |  |  |  |
|  | 1. Önerilerimle ilgili geri bildirim alamıyorsam |  |  |  |  |  |
|  | 1. Değişim olacağına inancım yoksa |  |  |  |  |  |
|  | 1. Kurumun bir parçası olmak istemiyorsam |  |  |  |  |  |
|  | 1. Konuşmaya gerek olmadığını düşünüyorsam |  |  |  |  |  |
|  | 1. Kurumu önemsememeye başladıysam |  |  |  |  |  |
|  | 1. İnsanlarla uğraşmaktan sıkıldıysam |  |  |  |  |  |
|  | 1. Kuruma bağlılığım azalmışsa |  |  |  |  |  |
|  | 1. Değersizleştirildiğimi düşünüyorsam |  |  |  |  |  |
| **Kurumu Korumaya Dayalı Sessizlik** | 1. Yetersizliğimin kuruma zarar vereceğini düşünüyorsam |  |  |  |  |  |
|  | 1. Teknolojik alt yapı ve donanım eksikliği varsa |  |  |  |  |  |
|  | 1. Kurumun rekabet gücünün azalacağını düşünüyorsam |  |  |  |  |  |
|  | 1. Kurumun itibarına zarar vereceğini düşünüyorsam |  |  |  |  |  |
|  | 1. Kurumun yeni yatırımlarıyla ilgili gizli bilgilere sahipsem |  |  |  |  |  |

Ölçek 4 alt boyuttan oluşmaktadır. Yukarıda alt boyutların dağılımı belirtilmiştir.

**Ölçek puanlaması**

Toplam ortalama puan üzerinden hesaplanmakta olup puan arttıkça sessizlik düzeyi artmaktadır.

Örneğin puan ortalaması 5’e yaklaştıkça sessizlik düzeyi yüksek olarak değerlendirilmektedir.
